# Prognostic Performance of Alternative Lymph Node Classification Systems for Patients with Medullary Thyroid Cancer: A Single Center Cohort Study

**DOI:** 10.1245/s10434-021-11134-3

**Published:** 2021-12-10

**Authors:** Dimitrios Prassas, Aristodemos Kounnamas, Kenko Cupisti, Matthias Schott, Wolfram Trudo Knoefel, Andreas Krieg

**Affiliations:** 1 Department of Surgery (A), Heinrich-Heine-University and University Hospital Duesseldorf, Duesseldorf, Germany; 2 Division for Specific Endocrinology, Heinrich-Heine-University and University Hospital Duesseldorf, Duesseldorf, Germany; 3 Department of Surgery, Marien-Hospital Euskirchen, Euskirchen, Germany

## Abstract

**Background:**

Lymph node ratio (LNR) and the log odds of positive lymph nodes (LODDS) have been proposed as alternative lymph node (LN) classification schemes. Various cut-off values have been defined for each system, with the question of the most appropriate for patients with medullary thyroid cancer (MTC) still remaining open. We aimed to retrospectively compare the predictive impact of different LN classification systems and to define the most appropriate set of cut-off values regarding accurate evaluation of overall survival (OS) in patients with MTC.

**Methods:**

182 patients with MTC who were operated on between 1985 and 2018 were extracted from our medical database. Cox proportional hazards regression models and C-statistics were performed to assess the discriminative power of 28 LNR and 28 LODDS classifications and compare them with the N category according to the 8th edition of the AJCC/UICC TNM classification in terms of discriminative power. Regression models were adjusted for age, sex, T category, focality, and genetic predisposition.

**Results:**

High LNR and LODDS are associated with advanced T categories, distant metastasis, sporadic disease, and male gender. In addition, among 56 alternative LN classifications, only one LNR and one LODDS classification were independently associated with OS, regardless of the presence of metastatic disease. The C-statistic demonstrated comparable results for all classification systems showing no clear superiority over the N category.

**Conclusion:**

Two distinct alternative LN classification systems demonstrated a better prognostic performance in MTC patients than the N category. However, larger scale studies are needed to further verify our findings.

**Supplementary Information:**

The online version contains supplementary material available at 10.1245/s10434-021-11134-3.

Medullary thyroid cancer (MTC) is a rare neuroendocrine malignancy that derives from the parafollicular cells or C-cells of the thyroid gland, accounting for approximately 1–2% of thyroid cancers.^1^ The primary curative treatment is complete resection of the tumor and its locoregional lymph node (LN) metastases. Total thyroidectomy is the standard treatment for MTC, as multifocal disease is present in up to 10% of patients with MTC and in nearly all patients with the inherited form of the disease.^1^ Prophylactic dissection of the central lymph node compartment is routinely performed in cases with no preoperative evidence of lymph node involvement, contrary to those with known neck and cervical LN metastases, where dissection of the involved lateral neck compartment is mandatory. Even if there is no evidence for contralateral metastases in diagnostic imaging in these cases, the contralateral compartment should also be resected if the basal serum calcitonin (Ctn) level is greater than 200 pg/ml. Postoperative monitoring of serum carcinoembryonic antigen (CEA) and serum Ctn levels provide valuable information about the potential presence of residual disease.^2,3^ As more data become available, it has been shown that dynamic risk stratification with adjusted response to initial therapy could offer more useful prognostic information than anatomic staging systems in MTC. This has been incorporated in the 2015 revised American Thyroid Association (ATA) guidelines where it is stated that TNM classification and postoperative serum Ctn level should be taken into account when predicting outcomes and planning long-term follow-up for patients treated by thyroidectomy for MTC.^1^ Thus, the present widely accepted LN classification system in use, as incorporated in the tumor node metastasis (TNM) system, might not adequately predict cases with residual disease and strongly depends on the extent of lymphadenectomy as well as the lymph node yield. This inherent weakness has paved the way for the development of alternative LN classification systems that have already been evaluated with regard to their prognostic ability in various malignant entities. The metastatic lymph node ratio (LNR) was the first to be suggested and is essentially the ratio of metastatic LNs to the total number of LNs harvested. A further system of alternative LN classification is the log odds of positive lymph nodes (LODDS) which is the natural logarithm of positive to negative LN ratio. Both LNR and LODDS have been found to be independent prognostic factors for disease-free survival in MTC.^4,5^ Nevertheless, profound diversity exists regarding cut-off points used to stratify the patient population. To date, no direct comparison has been performed in order to validate the prognostic ability of various LNR and LODDS cut-off points. In the present work, we sought to identify the most appropriate alternative LN classification system and compared the prognostic power of various cut-off points in patients with MTC that were treated in our department.

## Methods

### Study Population

Data from patients with MTC diagnosed between 1985 and 2018 were retrieved from the prospectively maintained computer-based patient records database of the University Hospital Duesseldorf. The final cohort analyzed by the study consisted of 182 patients. Cancer-specific and demographic data were retrieved for each patient. Surgical procedures for patients with MTC were performed as reported by Cupisti et al.^6^ and are summarized in Table 1. All included patients remained under outpatient follow-up. The study was performed in accordance with the principles of good clinical practice and the Declaration of Helsinki. An institutional review board approval of the Medical Faculty, Heinrich-Heine University Duesseldorf was retrieved (IRB-Nr: 2019-428ProspDEuA). Moreover, the study was registered in the German Clinical Trials Register (DRKS00021267). The present work adheres to the Standards for Reporting Diagnostic Accuracy Studies (STARD).^7^

**Table 1:** Patient characteristics

Variable	Overall
Number of subjects	182
*Age*	
Median (range)	50 (6-79)
*Gender n (%)*	
Male	75 (41.2)
Female	107 (58.8)
*Genetics n (%)*	
Sporadic	99 (54.4)
Familial (MEN2/FMTC)	62 (34.1)
Unknown	1 (0.5)
*Surgery type n (%)*	
Less than Tx	1 (0.5)
Tx + central LD (without lateral LD)	10 (5.5)
Tx + central LD + tumor side lateral LD	62 (34.1)
Tx + central LD + bilateral modified LD	60 (33.0)
+ mediastinal LD	45 (24.7)
+ liver resection	4 (2.2)
*Side affected n (%)*	
Unilateral	160 (87.9)
Bilateral	20 (11)
Unknown	2 (1.1)
*Focality n (%)*	
Unicentric	140 (22.5)
Multicentric	41 (36.5)
Unknown	1 (0.5)
*T category n (%)*	
T1 + T2	125 (68.7)
T3 + T4	57 (31.3)
*N category n (%)*	
N0	63 (34.6)
N1	119 (65.4)
*M category n (%)*	
M0	138 (75.8)
M1	44 (24.2)
Number of examined LNs, median (range)	15.5 (1-114)
Number of positive LNs, median (range)	2 (0-47)

Abbreviations: *Tx* = total thyroidectomy; *LD* = lymph node dissection, *LN* = lymph node

### Tumor Staging and LN Classifications

The TNM classification of malignant tumors 8th edition^8^ was utilized to define tumor stage. Cases already staged with an older edition were re-staged accordingly. The 8th edition system classifies LN involvement as N0 in the absence of regional metastases and N1 in the case of LN metastatic spread. LNR was calculated as the number of metastatic LNs harvested divided by the total number of examined LNs (NELN). LODDS was calculated using the following formula: log[(number of positive LNs + 0.5 )/(NELN - number of positive LNs + 0.5)]. LODDS and LNR were analyzed as both continuous and categorical variables. When used as categorical variables, cut-off values were defined by 40 previously published studies for 28 LNR^4,9–35^ and 28 LODDS^5,9,10,12,14–16,18–21,23,24,27,28,32,33,36–46^ classifications, respectively. Accordingly, each of the included LNR and LODDS classifications consisted of 2–5 subcategories. A literature search regarding available LNR as well as LODDS classifications was performed in January 2021.

### Statistical Analysis

The relationship between LN classification systems was initially explored with scatter plots. Receiver operating characteristics curves (ROC) were constructed in order to assess the accuracy of various LN classifications by measuring the area under the curve (AUC). The SPSS statistics software (IBM SPSS Statistics for Windows, Version 25.0. Armonk, NY: IBM Corp.) was used. Kaplan-Meier curves were generated and compared by the log-rank (Mantel-Cox) test using GraphPad Prism for Windows (Version 8.0.2, GraphPad Software, San Diego, California, USA). A multivariate Cox proportional regression model calculating hazard ratio (HR) and 95% confidence interval (CI) was used in order to analyze the relationship between distinct cut-off values of the different LN classifications and overall survival (OS). Accordingly, a base model was fitted including the following covariates: age, gender, T category (T1+2 vs T3+4), genetic background (familial vs sporadic form), and tumor focality (unifocal, multifocal). On the basis of the above-mentioned model, we evaluated each LN classification model discrimination using the concordance statistic (C-statistic), as recently described.^47^ A subgroup of patients without distal metastases was also analyzed. Differences in C-statistics were estimated by calculating the jackknife variance estimates of their difference. This is achieved by interpreting the 95% CI of the difference of the C-statistics. The delta C parameter was used to estimate the difference between N category and any other given parameter. Adjustment of the *p*-values of the above-mentioned comparison was performed by utilizing the false discovery rate (FDR).

In the event of missing data, a simple imputation method was utilized using medians for continuous variables and the commonest frequency for categorical outcomes.

The statistical software R version 3.6.3.^48^ was used for statistical analysis. We employed reporting tools using the R package “knitr”.^49^ The R’s package “survival”^50^ was used for the analysis based on the proportional hazard Cox regression and the calculation of the C-statistics.

## Results

**Fig. 1:**
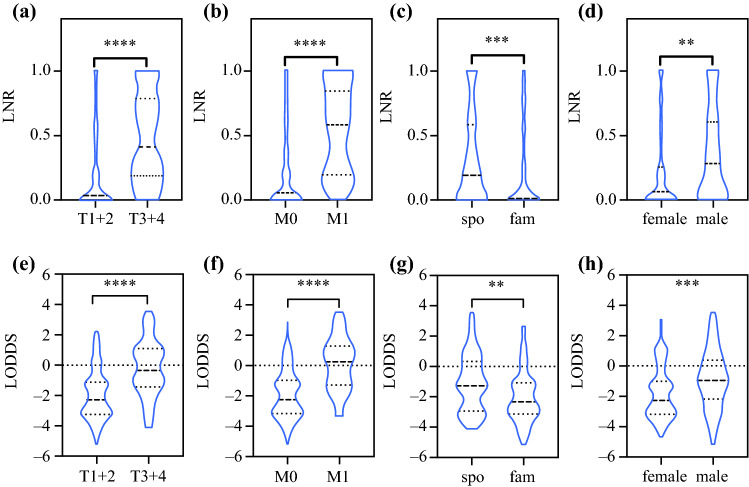
Association of LNR and LODDS with T category (**a** and **e**), presence of distant metastasis (**a** and **f**), genetic background (**c** and **g**) and gender (**d** and **h**). **p < 0.01; ***p < 0.001; ****p < 0.0001

The present study included a total of 182 cases with MTC. Baseline clinicopathological characteristics are depicted in Table 1. The study population consisted of 75 (41.2%) males and 107 (58.8%) females. The median age was 50 years (range 6–79 years). There were found to be 140 (22.5%) patients with unifocal and 41 (36.5%) patients with multifocal disease. Sixty-two (34.1%) patients were diagnosed with a familial form. Information with regard to ethnicity could not be retrospectively retrieved. The distribution of LNR and LODDS values in relation to assorted clinicopathologic variables and their subcategories was explored by creating violin plots (Fig. 1). Both high LNR and LODDS associated significantly with advanced T categories, distant metastasis, sporadic disease, and male gender.

**Fig. 2:**
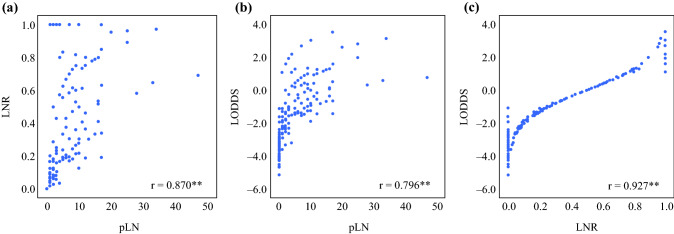
Scatter plot of the distribution of (**a**) LNR in relation to positive lymph nodes (pLN), (**b**) LODDS in relation to pLN and (**c**) LODDS in relation to LNR. Both LNR and LODDS increase with the number of pLN (r_s_ = 0.870, r_s_ = 0.796). Moreover, LODDS also increases with LNR values (r_s_ = 0.927). However, when LNR is 0 or 1, LODDS remains heterogeneous, implying that LODDS discriminates more precisely among patients without lymph node metastasis and patients in which the number of pLN is equal to the NELN

**Fig. 3:**
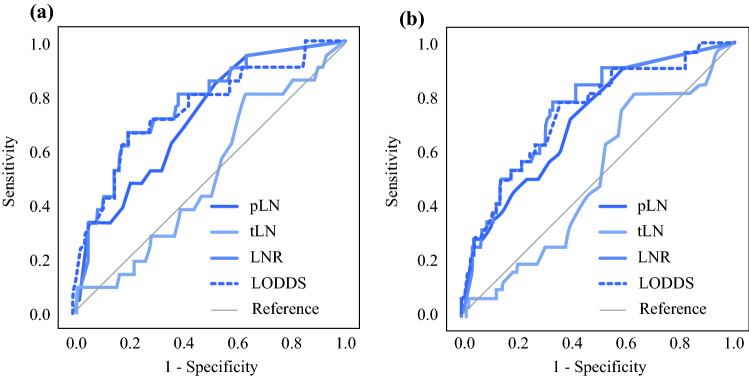
ROC analysis of LNR, LODDS, pLN, and tLN for (**a**) 5-year OS, (**b**) 10-year OS. LNR demonstrated the highest AUC values for both 5- and 10-year OS

**Fig. 4:**
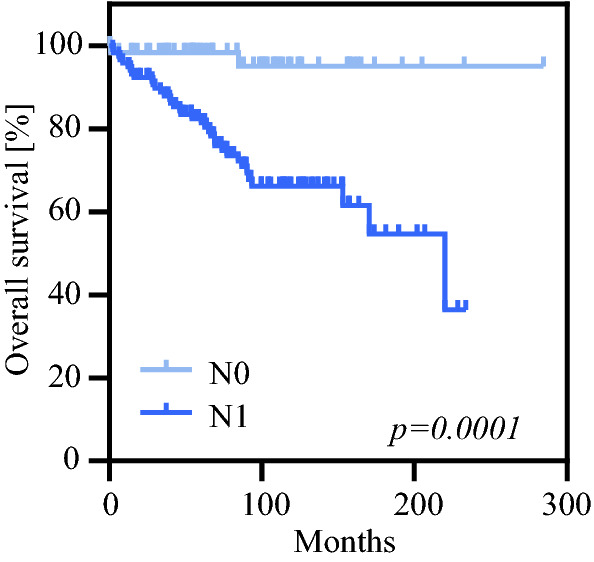
Kaplan Meier overall survival curves depending on the N category

First, LN parameters such as positive LNs (pLN), LNR, and LODDS were displayed in a scatter plot to explore any relationship between them (Fig. 2). LNR **(**Fig. 2a**)** and LODDS **(**Fig. 2b**)** increased parallel to the number of pLN (*r*_s_ = 0.870 and 0.796, respectively). In addition, LODDS was found to increase with LNR values (*r*_s_ = 0.927) **(**Fig. 2c**)**. Next, we performed a ROC analysis for 5-year and 10-year OS (Fig. 3a, b) and estimated the AUC. Interestingly, LNR demonstrated the highest AUC values for both 5- and 10-year OS (Table S1). To further elucidate the prognostic significance of the current N category as well as recently published alternative 28 LNR and 28 LODDS classification systems, we generated Kaplan-Meier survival curves demonstrating for all LN classification systems a significant association with OS (Fig. 4, S1, S2).

**Fig. 5:**
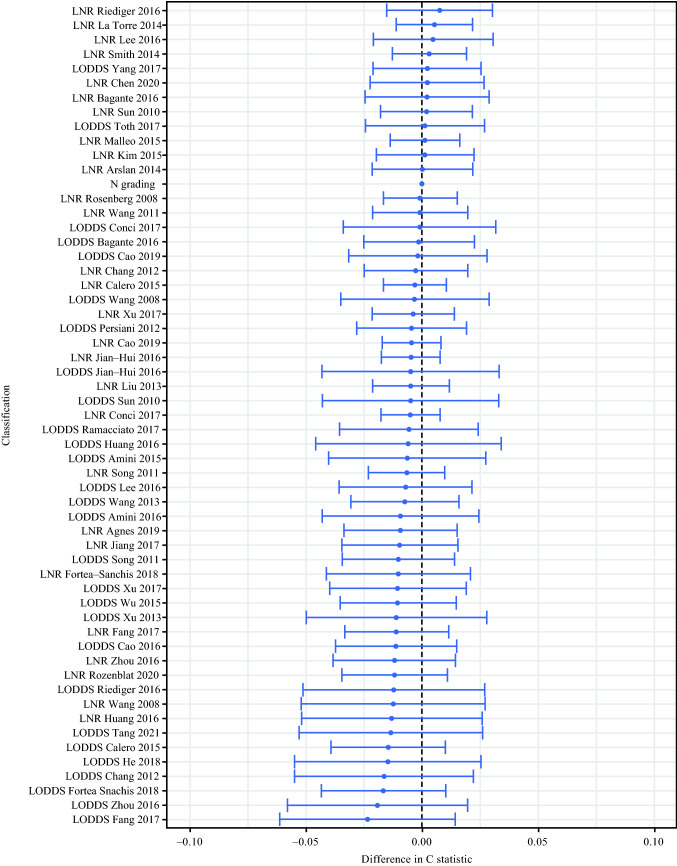
C-statistic comparing all alternative LN classifications to the N category (all patients). No LN classification scheme was found to out-perform the N category with regard to discriminatory power

To explore a potential superiority of various LNR or LODDS classifications over the currently used N category, Cox proportional hazards regression was performed with subsequent evaluation of model discrimination for each LN classification system using the C-statistic. Therefore, in a first step we examined the prognostic value of the selected covariates in a base model, initially for all included cases, using Cox regression analysis. Accordingly, age and presence of distant metastasis were significantly associated with OS (HR_age_ 2.53; 95% CI 1.10–5.80; *p* = 0.029; HR_M_ 4.74; 95% CI 2.26–9.95; *p* < 0.001) (Table 2). Using this base model, we performed in a second step Cox regression analysis for each LN classification system separately (Table S2 and 3) and, subsequently, assessed model discrimination by applying C-statistics in our entire cohort of MTC patients. Interestingly, the N category failed to reach statistical significance regarding OS in the entire group of MTC patients (HR 3.11; 95% CI 0.66–14.77; *p* = 0.153). In contrast, out of the 28 LNR and 28 LODDS classification systems, LNR classification as proposed by Chen and colleagues^34^ and LODDS classification by Ramacciato et al.^38^ were the only ones to demonstrate a significant association between increasing LNR or LODDS categories and poor prognosis. It should be mentioned that there were no cases in the LODDS 3 category as defined by Ramacciato et al.^38^ However, none of these alternative LN classification systems provided a better prognostic discrimination when compared with the classic N category (Table S2 and 3) (Fig. 5).

**Table 2:** Multivariate Cox regression analysis of the variables considered for the adjusted base model

	All patients		Non–metastatic patients (M0)	
Risk factor	HR (95% CI)	*p*-value	HR (95% CI)	*p*-value
*Age*				
Age<50	1.00 (reference)	0.029	1.00 (reference)	0.025
Age≥50	2.58 (1.10-5.80)		6.90 (1.27-37.38)	
*Gender*				
Male	1.00 (reference)	0.759	1.00 (reference)	0.706
Female	1.11 (0.57-2.14)		1.26 (0.38-5.00)	
*T stage*				
T3 + T4	1.00 (reference)	0.235	1.00 (reference)	0.381
T1 + T2	0.65 (0.32-1.33)		1.89 (0.45-7.90)	
*MEN syndrome*				
Yes	1.00 (reference)	0.790	1.00 (reference)	0.374
No	1.15 (0.41-3.27)		2.19 (0.39-12.40)	
*Genetic status*				
Known	1.00 (reference)	0.144	1.00 (reference)	0.304
Unknown	2.05 (0.78-5.35)		2.48 (0.44-13.96)	
*Tumor multifocality*				
Yes	1.00 (reference)	0.087	1.00 (reference)	0.222
No	0.41 (0.15-1.14)		0.33 (0.05-1.97)	
*M stage*				
M0	1.00 (reference)	<0.001	N/A	
M1	4.74 (2.26-9.95)

Since we were able to show that distant metastasis correlated with poor survival, we decided in a next step to examine the prognostic value of the various LN classifications only in the subgroup of patients without distant metastases (M0). In fact, in addition to the aforementioned classifications, in the M0 subgroup further classifications^5,9–15,17–19,22–28,31–36,38–40,43^ also demonstrated an association between increasing LNR/LODDS subcategories and OS (Table S2 and 3). Note also that the currently used N category was significantly associated with a poor prognosis in the M0 subgroup (HR 9.971; 95% CI 1.172–84.851; *p* = 0.035). However, again none of these alternative LN classifications demonstrated superior prognostic discrimination over the N category defined by the AJCC/UICC 8th edition.

## Discussion

Novel LN classification systems such as LNR and LODDS have been developed as an alternative to the widely used N category, in order to overcome the inherent weaknesses of the latter. LNR was developed essentially as an LN classification system that takes into account the radicality of lymphadenectomy, a characteristic that is not entirely reflected in the currently used N category, as it is independent of the total number of harvested LNs. Nevertheless, LNR demonstrates limited predictive power in cases where retrieved LNs were either all positive (LNR = 1) or all negative (LNR = 0). Therefore, LODDS was introduced as an LN classification system that can more precisely reflect the extent of radicality and further sub-stratify cases even with LNR values of 0 or 1. Very few studies have investigated the prognostic value of alternative LN classifications regarding MTC. Rozenblat et al.,^4^ Chen et al.,^34^ as well as Jiang et al.^35^ have already demonstrated the discriminative power of LNR for MTC, whereas Tang and colleagues^5^ reached the same results with regard to LODDS. Both of the studied novel LN classification systems, LNR and LODDS, are continuous biological variables. Such a variable format is deemed unsuitable for clinical use, as distinct classification subgroups are necessary. A plentitude of different cut-off points has been proposed for various types of cancer entities, with the most appropriate ones for MTC yet to be decided. In the present study, 28 LNR and 28 LODDS classifications were investigated in a cohort of 182 patients following surgery for MTC in our department.

The measured AUC demonstrated similar accuracy for both alternative LN classifications, both being superior to the pLN, hence validating their predictive value in our patient cohort when used as a continuous variable. Subsequently, we aimed to compare LNR and LODDS as categorical variables, based on already published cut-off points and defined subcategories. Cox regression analysis was undertaken for both patient groups with and without distant metastasis. Apart from age, various LN classifications were found to be independent prognostic factors for OS in both patient subgroups. Nonetheless, a classification scheme meets it purpose not only when it is statistically significant, but also when its subcategories are characterized by linearity regarding their HRs. In other words, each higher subcategory of a given classification should be associated with worse outcome than the previous, in terms of HR. Between all investigated LN classifications, the only classifications that were found to satisfy the above-mentioned criteria were LNR and LODDS as proposed by Chen et al.^34^ and Ramacciato et al.^38^, respectively, regardless of the presence of distant metastasis. Moreover, when a multivariate analysis approach was performed for the whole patient collective, LNR of Chen et al.^34^ and LODDS defined by Ramacciato et al.^38^ were the only LN classifications that were found to be independent prognostic factors for OS, while N category was not. Overall, four studies address the issue of the most appropriate alternative LN classification scheme specifically in MTC patients.^4,5,34,35^ Not only did Chen and colleagues^34^ define cut-off values for LNR in the largest MCT collective (*n* = 1237), but also the analysis focused exclusively on OS. Tang and co-workers^5^ also defined cut-off values for LODDS subcategories in a large MTC population, nevertheless focusing on DFS rather than OS. Jiang et al.^35^ and Rozenblat et al.^4^ used a relatively small patient population to define their cut-off values (416 and 107 MTC cases respectively). At this point, it can be safely stated that any future attempts to define novel cut-off values should be made in the setting of large MTC patient collectives for both OS and DFS.

Furthermore, we attempted to compare the discriminative capacity of the 56 above-mentioned various LNR and LODDS classifications^4,5,9–46^ with the contemporary N category by means of the C-statistic. The C-index was calculated for each regression model, using the one containing the LN classification as the reference model. However, none of the alternative LN classification schemes was found to be significantly superior to the N category in our study cohort, even after excluding cases with distant metastasis.

Our study still has some limitations as the cohort size was not exceptionally large. In addition, a possible explanation for our ambiguous findings might be the low absolute number of events during the follow-up (deaths, *n* = 37) that reduces the sensitivity of our analysis. Second, biochemical factors of proven prognostic relevance, such as Ctn and CEA serum levels, could not be retrieved for all patients from our database and subsequently could not be incorporated in our investigation. Third, the patients represent a selected cohort treated in a highly specialized setting and are therefore not representative of all patients diagnosed with MTC. Finally, the retrospective design of our study and the fact that we investigated OS only and not DFS represents a further weakness of the present work.

However, to the best of our knowledge, this is the first work to compare a large set of previously published LN classification systems in predicting survival in patients with MTC. We performed an extensive analysis of various LN classifications and generated novel data that lay the groundwork for future research, pointing the focus upon a distinct set of LNR and LODDS cut-off values as proposed by Chen et al.^34^ and Ramacciato et al.^38^ respectively, that seem to be clinically relevant. This constitutes a considerable finding that should be further evaluated through larger and multicenter cohort studies in patients with MTC.

## Conclusion

In conclusion, LNR and LODDS as proposed by Chen et al.^34^ and Ramacciato et al.^38^ respectively, were identified as alternative LN classifications that might be most suitable for cases with MTC, regardless of the presence of metastatic disease, in terms of predicting OS. However, none of the investigated novel LN classification systems demonstrated clear discriminative superiority in the prediction of prognosis over the currently implemented N category in MTC patients. This work provides an important foundation for future research in the context of carefully designed larger scale clinical trials.

## Supplementary Information

Below is the link to the electronic supplementary material.

Supplementary file1 (DOCX 911 KB)
