# Supplementary material for: Prognostic Performance of Alternative Lymph Node Classification Systems for Patients with Medullary Thyroid Cancer: A Single Center Cohort Study
Source: Ann Surg Oncol. 2021 Dec 10;29(4):2561–9. doi: 10.1245/s10434-021-11134-3 (PMC8933356; doi:10.1245/s10434-021-11134-3)
Supplement: Supplementary file 1 — Supplementary file1 (DOCX 911 KB) [file 10434_2021_11134_MOESM1_ESM.docx]

**FIG. S1** Kaplan-Meier survival curves for OS depending on the LNR classification system reported by **(a)** Xu et al.^12^, **(b)** Arslan et al.^11^, **(c)** Malleo et al.^13^, **(d)** Sun et al.^10^, **(e)** Zhou et al.^14^, **(f)** Riediger et al.^15^, **(g)** Fang et al.^16^, **(h)** Wang et al.^17^, **(i)** Conci et al.^18^, **(j)** Huang et al.^19^, **(k)** Calero et al.^20^, **(l)** Lee et al.^21^, **(m)** Kim et al.^22^, **(n)** Bagante et al.^23^, **(o)** Jian-Hui et al.^24^, **(p)** Smith et al.^25^, **(q)** Liu et al.^26^, **(r)** Chang et al.^27^, **(s)** Song et al.^28^, **(t)** La Torre et al.^29^, **(u)** Agnes et al.^30^, **(v)** Rosenberg et al.^31^, **(w)** Cao et al.^32^, **(x)** Fortea-Sanchis et al.^9^, **(y)** Wang et al.^33^, **(z)** Chen et al.^34^, **(aa)** Rozenblat et al.^4^, **(ab)** Jiang et al.^35^


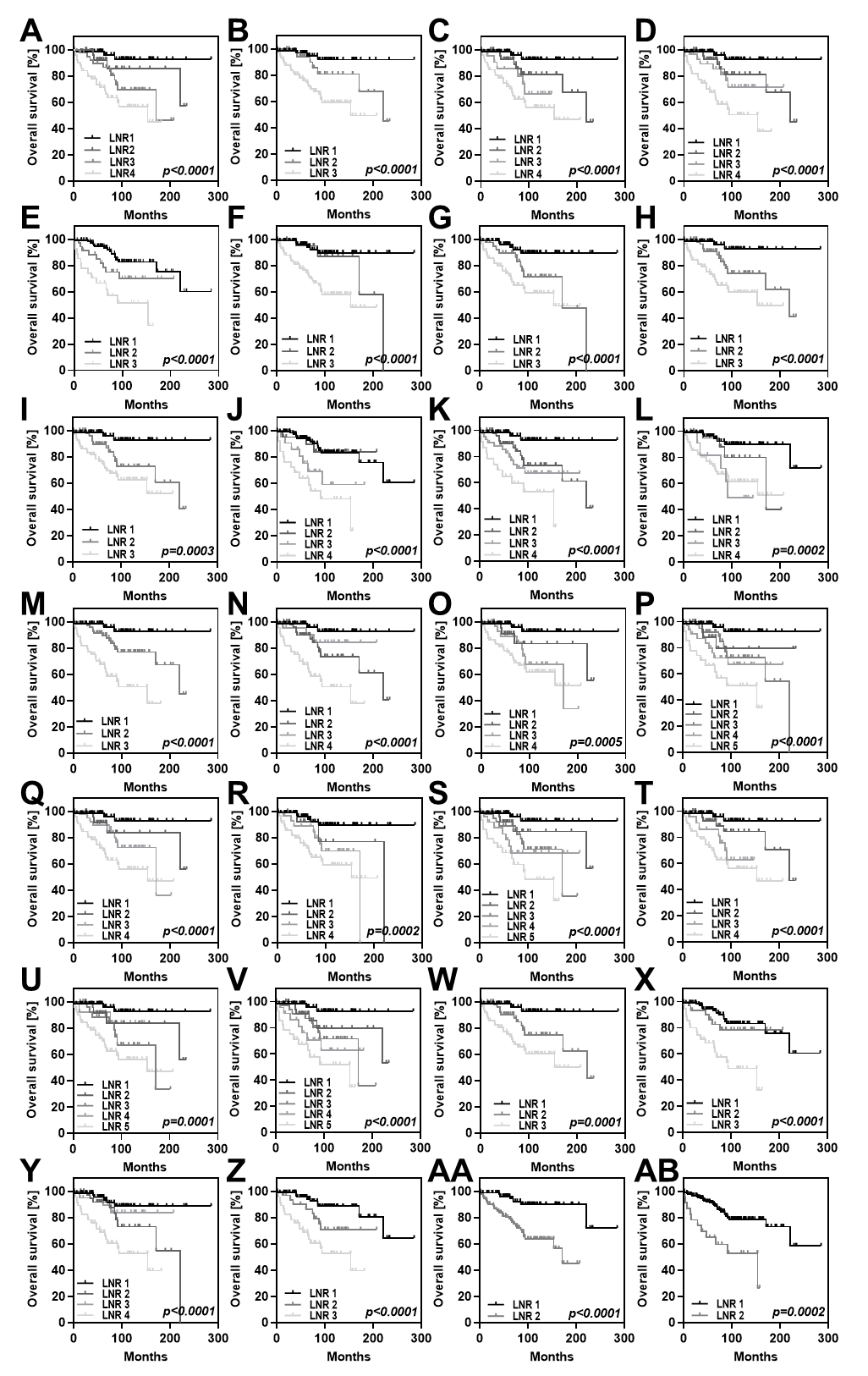


**FIG. S2** Kaplan-Meier survival curves for OS depending on the LODDS classification system reported by **(a)** Xu et al.^12^, **(b)** Yang et al.^36^, **(c)** Fortea-Sanchis et al.^9^, **(d)** He et al.^37^, **(e)** Ramacciato et al.^38^, **(f)** Toth et al.^39^, **(g)** Zhou et al.^14^, **(h)** Sun et al.^10^, **(i)** Xu et al.^40^, **(j)** Riediger et al.^15^, **(k)** Fang et al.^16^, **(l)** Conci et al.^18^, **(m)** Huang et al.^19^, **(n)** Calero et al.^20^, **(o)** Lee et al.^21^, **(p)** Cao et al.^32^, **(q)** Amini et al.^42^, **(r)** Bagante et al.^23^, **(s)** Jian-Hui et al.^24^, **(t)** Wu et al.^43^, **(u)** Wang et al.^44^, **(v)** Chang et al.^27^, **(w)** Song et al.^28^, **(x)** Persiani et al.^45^, **(y)** Wang et al.^33^, **(z)** Cao et al.^32^, **(aa)** Amini et al.^46^, **(ab)** Tang et al.^5^

**
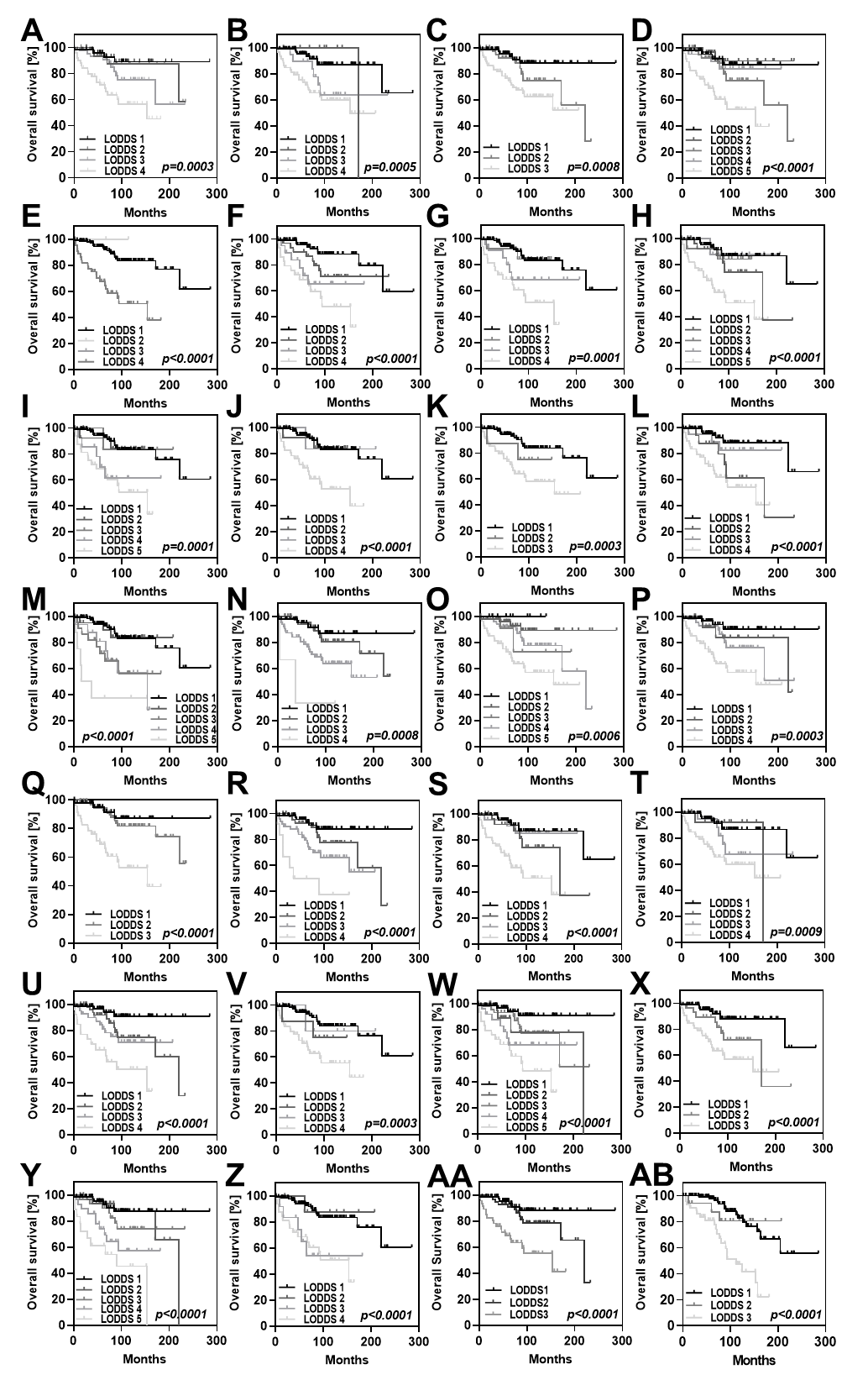
**

**FIG. S3** C-statistic demonstrating comparable results for all classification systems showing no superiority of any LNR or LODDS classification when compared to the N category in patients without distant metastasis


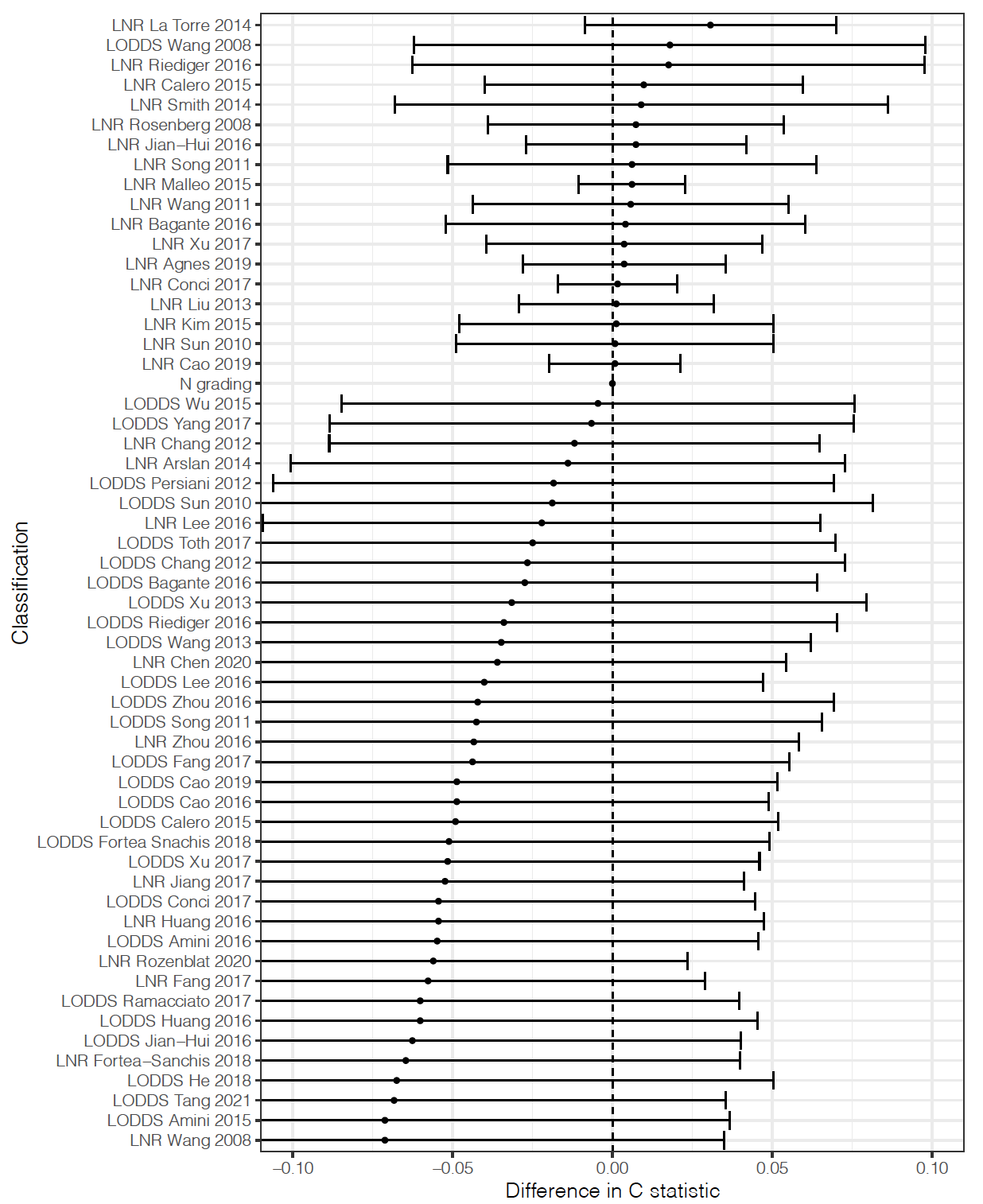


| **TABLE S1** ROC analysis of the total number of resected lymph nodes (tLN), positive lymph nodes (pLN), LNR and LODDS. for 5- year, and 10-year OS | | | | |
| --- | --- | --- | --- | --- |
| Overall Survival (OS) | LN evaluation | ROC analysis | | |
|  | Type | AUC | 95% CI | P value |
| *5-year OS* | LODDS | 0.752 | 0.632-0.873 | **< 0.001** |
|  | LNR | 0.766 | 0.659-0.873 | **< 0.001** |
|  | tLN | 0.506 | 0.380-0.633 | 0.926 |
|  | pLN | 0.716 | 0.606-0.826 | **0.002** |
| *10-year OS* | LODDS | 0.744 | 0.650-0.839 | **< 0.001** |
|  | LNR | 0.753 | 0.663-0.843 | **< 0.001** |
|  | tLN | 0.505 | 0.402-0.609 | 0.923 |
|  | pLN | 0.714 | 0.619-0.809 | **< 0.001** |

| **TABLE S2.** OS depending on the respective LNR classification. Each LNR subgroup is defined by a LNR range as indicated | | | | | | | | | |
| --- | --- | --- | --- | --- | --- | --- | --- | --- | --- |
|  |  |  | *All patients* | | | | *Non-metastatic (M0) patients* | | |
| LNR Classification | Subgroup | LNR Range | HR | 95% CI | P value | HR | | 95%CI | P value |
| Agnes et al^30^ | 1 | 0 | 1 | (Reference) |  | 1 | | (Reference) |  |
|  | 2 | 0.01; 0.10 | 1.841 | 0.363-9.339 | 0.461 | 10.656 | | 1.080-105.115 | **0.042** |
|  | 3 | 0.11; 0.25 | 1.905 | 0.446-8.145 | 0.385 | 5.806 | | 0.439-76.780 | 0.128 |
|  | 4 | 0.26; 0.40 | 0.718 | 0.089-5.811 | 0.756 | 8.271 | | 0.269-254.017 | 0.226 |
|  | 5 | >0.40 | 2.343 | 0.560-9.817 | 0.244 | 12.159 | | 1.146-128.926 | **0.038** |
| Arslan et al^11^ | 1 | ≤0.05 | 1 | (Reference) |  | 1 | | (Reference) |  |
|  | 2 | >0.05-0.20 | 1.126 | 0.292-4.336 | 0.863 | 3.690 | | 0.584-23.339 | 0.165 |
|  | 3 | >0.20 | 2.248 | 0.622-8.122 | 0.217 | 7.810 | | 1.272-47.927 | **0.026** |
| Bagante et al^23^ | 1 | 0 | 1 | (Reference) |  | 1 | | (Reference) |  |
|  | 2 | 0.01; 0.25 | 1.992 | 0.513-7.741 | 0.319 | 8.269 | | 0.883-77.400 | **0.061** |
|  | 3 | 0.26; 0.50 | 0.719 | 0.115-4.500 | 0.724 | 3.462 | | 0.173-69.677 | 0.417 |
|  | 4 | >0.50 | 6.794 | 3.949-11.688 | 0.135 | 22.581 | | 2.161-235.972 | **0.009** |
| Calero et al^20^ | 1 | 0 | 1 | (Reference) |  | 1 | | (Reference) |  |
|  | 2 | 0.01; 0.25 | 1.977 | 0.509-7.685 | 0.325 | 8.624 | | 0.921-80.591 | 0.059 |
|  | 3 | 0.26; 0.75 | 1.830 | 0.405-8.277 | 0.433 | 8.354 | | 0.699-99.793 | 0.093 |
|  | 4 | >0.75 | 3.019 | 0.708-12.865 | 0.166 | 52.482 | | 3.706-743.141 | **0.003** |
| Cao et al^32^ | 1 | 0; 0.24 | 1 | (Reference) |  | 1 | | (Reference) |  |
|  | 2 | 0.25; 0.28 | 1.910 | 0.492-7.421 | 0.350 | 8.237 | | 0.907-74.789 | 0.061 |
|  | 3 | >0.28 | 2.353 | 0.560-9.888 | 0.243 | 14.851 | | 1.470-150.023 | **0.022** |
| Chang et al^27^ | 1 | ≤0.08 | 1 | (Reference) |  | 1 | | (Reference) |  |
|  | 2 | 0.09; 0.17 | 0.581 | 0.119-2.852 | 0.504 | 9.529 | | 0-Inf | 0.998 |
|  | 3 | 0.18; 0.33 | 1.506 | 0.417-5.433 | 0.531 | 12.417 | | 0.614-33.990 | 0.138 |
|  | 4 | >0.33 | 1.480 | 0.429-5.113 | 0.535 | 12.129 | | 0.863-23.070 | 0.074 |
| Chen et al^34^ | 1 | <0.19 | 1 | (Reference) |  | 1 | | (Reference) |  |
|  | 2 | 0.19; 0.49 | 1.614 | 0.526-4.957 | 0.403 | 3.108 | | 0.455-21.240 | 0.247 |
|  | 3 | >0.49 | 2.707 | 1.041-7.038 | **0.041** | 7.782 | | 1.633-37.095 | **0.010** |
|  |  |  |  |  |  |  | |  |  |
|  |  |  |  |  |  |  | |  |  |
| Conci et al^18^ | 1 | 0 | 1 | (Reference) |  | 1 | | (Reference) |  |
|  | 2 | >0; ≤0.25 | 1.941 | 0.499-7.555 | 0.339 | 8.616 | | 0.952-77.956 | 0.055 |
|  | 3 | >0.25 | 2.253 | 0.538-9.434 | 0.266 | 13.551 | | 1.325-138.619 | **0.028** |
| Fang et al^16^ | 1 | <0.1 | 1 | (Reference) |  | 1 | | (Reference) |  |
|  | 2 | 0.1; 0.33 | 1.245 | 0.400-3.875 | 0.706 | 1.978 | | 0.321-12-117 | 0.462 |
|  | 3 | ≥0.34 | 1.603 | 0.507-5.071 | 0.422 | 3.640 | | 0.704-18.821 | 0.123 |
| Fortea-Sanchis et al^9^ | 1 | 0; 0.24 | 1 | (Reference) |  | 1 | | (Reference) |  |
|  | 2 | 0.25; 0.60 | 0.712 | 0.234-2.219 | 0.567 | 1.262 | | 0.198-8.062 | 0.805 |
|  | 3 | >0.60 | 1.724 | 0.713-4.172 | 0.227 | 5.928 | | 1.354-25.944 | **0.018** |
| Huang et al^19^ | 1 | <0.25 | 1 | (Reference) |  | 1 | | (Reference) |  |
|  | 2 | ≥0.25; <0.5 | 0.419 | 0.100-1.760 | 0.235 | 0.870 | | 0.083-9.146 | 0.907 |
|  | 3 | ≥0.50; <0.75 | 1.638 | 0.597-4.493 | 0.338 | 2.871 | | 0.511-16.104 | 0.231 |
|  | 4 | ≥0.75 | 1.597 | 0.625-4.080 | 0.328 | 10.850 | | 1.757-66.988 | **0.010** |
| Jian-Hui et al^24^ | 1 | 0 | 1 | (Reference) |  | 1 | | (Reference) |  |
|  | 2 | 0.01; ≤ 0.1 | 1.942 | 0.382-9.881 | 0.424 | 10.918 | | 1.104-107.941 | **0.041** |
|  | 3 | >0.1; ≤ 0.25 | 1.936 | 0.455-8.237 | 0.371 | 5.796 | | 0.448-74.893 | 0.178 |
|  | 4 | >0.25 | 2.264 | 0.537-9.542 | 0.266 | 11.880 | | 1.112-126.958 | **0.041** |
| Jiang et al^35^ | 1 | 0; ≤0.765 | 1 | (Reference) |  | 1 | | (Reference) |  |
|  | 2 | ≥0.765 | 1.593 | 0.728-3.483 | 0.244 | 9.018 | | 1.700-47.903 | **0.010** |
| La Torre et al^29^ | 1 | 0 | 1 | (Reference) |  | 1 | | (Reference) |  |
|  | 2 | 0.010; 0.199 | 1.357 | 0.318-5.784 | 0.680 | 6.068 | | 0.607-60.582 | 0.124 |
|  | 3 | 0.200; 0.399 | 2.962 | 0.605-14.511 | 0.180 | 29.635 | | 2.112-415.828 | **0.012** |
|  | 4 | >0.399 | 3.140 | 0.764-12.906 | 0.113 | 18.194 | | 1.802-183.748 | **0.014** |
| Lee et al^21^ | 1 | ≤0.1 | 1 | (Reference) |  | 1 | | (Reference) |  |
|  | 2 | >0.1; ≤ 0.2 | 0.894 | 0.228-3.510 | 0.873 | 1.115 | | 0.103-12.053 | 0.929 |
|  | 3 | >0.2; ≤ 0.3 | 2.763 | 0.697-10.957 | 0.148 | 3.529 | | 0.290-42.869 | 0.322 |
|  | 4 | >0.3 | 1.816 | 0.590-5.588 | 0.298 | 4.246 | | 0.828-21.766 | 0.083 |
|  |  |  |  |  |  |  | |  |  |
|  |  |  |  |  |  |  | |  |  |
|  |  |  |  |  |  |  | |  |  |
|  |  |  |  |  |  |  | |  |  |
|  |  |  |  |  |  |  | |  |  |
| Liu et al^26^ | 1 | 0 | 1 | (Reference) |  | 1 | | (Reference) |  |
|  | 2 | 0.01; 0.10 | 1.931 | 0.383-1.749 | 0.426 | 10.755 | | 1.089-106.189 | **0.042** |
|  | 3 | 0.11; 0.40 | 1.615 | 0.378-6.888 | 0.518 | 6.173 | | 0.498-76.375 | 0.156 |
|  | 4 | >0.40 | 2.658 | 0.650-10.860 | 0.174 | 11.739 | | 1.120-123.013 | **0.039** |
| Malleo et al^13^ | 1 | 0 | 1 | (Reference) |  | 1 | | (Reference) |  |
|  | 2 | >0; ≤0.2 | 1.528 | 0.370-6.320 | 0.558 | 7.695 | | 0.817-72.471 | 0.075 |
|  | 3 | >0.2; 0.4 | 2.388 | 0.473-12.051 | 0.292 | 14.665 | | 0.955-225.157 | 0.054 |
|  | 4 | >0.4 | 2.956 | 0.720-12.134 | 0.133 | 14.974 | | 1.478-151.722 | **0.022** |
| Kim et al^22^ | 1 | 0 | 1 | (Reference) |  | 1 | | (Reference) |  |
|  | 2 | >0; ≤ 0.5 | 1.671 | 0.430-6.487 | 0.458 | 7.190 | | 0.781-66.177 | 0.082 |
|  | 3 | >0.5 | 3.537 | 0.851-14.699 | 0.082 | 24.425 | | 2.399-248.579 | **0.007** |
| Riediger et al^15^ | 1 | < 0.1 | 1 | (Reference) |  | 1 | | (Reference) |  |
|  | 2 | 0.1; 0.199 | 0.651 | 0.157-2.699 | 0.554 | 4.905 | | 0-Inf. | 0.998 |
|  | 3 | ≥0.2 | 2.004 | 0.639-6.287 | 0.233 | 4.444 | | 1.078-24.388 | **0.039** |
| Rosenberg et al^31^ | 1 | 0 | 1 | (Reference) |  | 1 | | (Reference) |  |
|  | 2 | 0.01; 0.17 | 1.555 | 0.354-6.830 | 0.559 | 7.260 | | 0.718-73.367 | 0.093 |
|  | 3 | 0.18; 0.41 | 1.994 | 0.440-9.033 | 0.370 | 12.178 | | 0.878-168.801 | 0.062 |
|  | 4 | 0.42; 0.69 | 2.754 | 0.618-12.282 | 0.184 | 11.379 | | 0.861-150.226 | 0.065 |
|  | 5 | ≥0.70 | 3.067 | 0.681-13.810 | 0.144 | 45.667 | | 3.129-666.429 | **0.005** |
| Rozenblat et al^4^ | 1 | ≤0.1 | 1 | (Reference) |  | 1 | | (Reference) |  |
|  | 2 | >0.1 | 1.530 | 0.535-4.371 | 0.427 | 2.882 | | 0.602-13.803 | 0.185 |
| Smith et al^25^ | 1 | 0 | 1 | (Reference) |  | 1 | | (Reference) |  |
|  | 2 | >0; ≤ 1/15 | 3.167 | 0.616-16.270 | 0.168 | 14.158 | | 1.318-152.038 | **0.028** |
|  | 3 | >1/15; ≤ 3/10 | 1.470 | 0.345-6.258 | 0.603 | 3.969 | | 0.297-53.016 | 0.297 |
|  | 4 | >3/10; ≤ 7/10 | 1.962 | 0.433-8.900 | 0.382 | 8.498 | | 0.711-101.576 | 0.090 |
|  | 5 | >7/10 | 2.629 | 0.576-12.012 | 0.212 | 33.972 | | 2.449-471.165 | **0.009** |
|  |  |  |  |  |  |  | |  |  |
|  |  |  |  |  |  |  | |  |  |
|  |  |  |  |  |  |  | |  |  |
|  |  |  |  |  |  |  | |  |  |
|  |  |  |  |  |  |  | |  |  |
| Song et al^28^ | 1 | 0 | 1 | (Reference) |  | 1 | | (Reference) |  |
|  | 2 | 0.01; 0.11 | 1.487 | 0.294-7.514 | 0.632 | 11.497 | | 1.141-115.838 | **0.038** |
|  | 3 | 0.12; 0.36 | 1.862 | 0.436-7.955 | 0.402 | 6.634 | | 0.534-82.403 | 0.141 |
|  | 4 | 0.37; 0.66 | 2.801 | 0.640-12.254 | 0.171 | 6.082 | | 0.445-83.138 | 0.176 |
|  | 5 | >0.66 | 2.746 | 0.603-12.505 | 0.192 | 35.256 | | 2.487-499.724 | **0.008** |
| Sun et al^10^ | 1 | 0 | 1 | (Reference) |  | 1 | | (Reference) |  |
|  | 2 | 0.01; 0.20 | 1.578 | 0.383-6.512 | 0.528 | 7.521 | | 0.780-72.535 | 0.081 |
|  | 3 | 0.21; 0.50 | 1.869 | 0.397-8.794 | 0.428 | 6.314 | | 0.454-87.851 | 0.170 |
|  | 4 | >0.50 | 3.640 | 0.864-15.331 | 0.078 | 23.943 | | 2.323-246.818 | **0.008** |
| Wang et al^17^ | 1 | 0 | 1 | (Reference) |  | 1 | | (Reference) |  |
|  | 2 | 0.01; 0.30 | 0.889 | 0.264-3.000 | 0.850 | 1.121 | | 0.181-8.088 | 0.843 |
|  | 3 | 0.31; 0.60 | 0.383 | 0.069-2.142 | 0.274 | 0.894 | | 0.068-11.732 | 0.932 |
|  | 4 | 0.61; 1 | 1.476 | 0.421-5.169 | 0.543 | 4.962 | | 0.972-25.316 | 0.054 |
| Wang et al^33^ | 1 | <0.07 | 1 | (Reference) |  | 1 | | (Reference) |  |
|  | 2 | 0.07; < 0.25 | 1.883 | 0.484-7.325 | 0.361 | 8.048 | | 0.853-75.908 | 0.068 |
|  | 3 | 0.25; < 0.50 | 1.616 | 0.324-8.058 | 0.559 | 9.022 | | 0.664-122.504 | 0.098 |
|  | 4 | 0.50; 1 | 3.379 | 0.782-14.592 | 0.103 | 30.375 | | 2.595-355.625 | **0.007** |
| Xu et al^12^ | 1 | 0 | 1 | (Reference) |  | 1 | | (Reference) |  |
|  | 2 | >0; ≤ 0.125 | 1.412 | 0.283-7.043 | 0.674 | 9.876 | | 0.994-98.042 | **0.050** |
|  | 3 | >0.125; ≤ 0.425 | 1.933 | 0.457-8.167 | 0.370 | 5.979 | | 0.480-74.403 | 0.165 |
|  | 4 | >0.425; ≤ 1 | 2.891 | 0.705-11.852 | 0.140 | 14.656 | | 1.406-152.775 | **0.025** |
| Zhou et al^14^ | 1 | 0; ≤0.30 | 1 | (Reference) |  | 1 | | (Reference) |  |
|  | 2 | >0.30; ≤0.70 | 1.106 | 0.440-2.776 | 0.830 | 2.040 | | 0.417-10.000 | 0.379 |
|  | 3 | >0.70; ≤1 | 1.639 | 0.678-3.962 | 0.272 | 8.946 | | 1.559-51.333 | **0.014** |

**TABLE S3** OS depending on the respective LODDS classification. Each LODDS subgroup is defined by a LODDS range as indicated. *No cases were identified under subgroup 3

|  |  |  | *All patients* | | | *Non-metastatic (M0) patients* | | |
| --- | --- | --- | --- | --- | --- | --- | --- | --- |
| LODDS Classification | Subgroup | LODDS Range | HR | 95%CI | P value | HR | 95%CI | P value |
| Amini et al^46^ | 1 | ≤ -2 | 1 | (Reference) |  | 1 | (Reference) |  |
|  | 2 | -2; <0 | 0.720 | 0.220-2.355 | 0.587 | 0.846 | 0.154-4.663 | 0.848 |
|  | 3 | ≥ 0 | 1.629 | 0.493-5.376 | 0.423 | 4.386 | 0.918-20.957 | 0.064 |
| Amini et al^42^ | 1 | ≤ -3 | 1 | (Reference) |  | 1 | (Reference) |  |
|  | 2 | >-3; <0 | 0.694 | 0.203-2.376 | 0.561 | 1.525 | 0.278-8.365 | 0.627 |
|  | 3 | ≥ 0 | 1.542 | 0.424-5.603 | 0.511 | 6.528 | 1.050-40.606 | **0.044** |
| Bagante et al^23^ | 1 | <-2 | 1 | (Reference) |  | 1 | (Reference) |  |
|  | 2 | -1.99; -0.90 | 0.908 | 0.277-2.982 | 0.874 | 0.947 | 0.155-5.783 | 0.953 |
|  | 3 | - 0.89; 1.50 | 1.120 | 0.323-3.879 | 0.858 | 1.993 | 0.342-11.615 | 0.443 |
|  | 4 | >1.5 | 2.611 | 0.580-11.751 | 0.211 | 40.978 | 4.823-348.163 | **0.001** |
| Calero et al^20^ | 1 | ≤-3 | 1 | (Reference) |  | 1 | (Reference) |  |
|  | 2 | >-3; ≤-1 | 0.809 | 0.236-2.781 | 0.737 | 1.673 | 0.230-9.342 | 0.557 |
|  | 3 | >-1; ≤3 | 1.074 | 0.289-3.989 | 0.916 | 4.232 | 0.657-27.247 | 0.129 |
|  | 4 | >3 | 1.761 | 0.237-13.110 | 0.581 | N/A | N/A | N/A |
| Cao et al^32^ | 1 | ≤-0.5 | 1 | (Reference) |  | 1 | (Reference) |  |
|  | 2 | >-0.5; ≤0 | 0.472 | 0.062-3.624 | 0.470 | 5.937 | 0-Inf. | 0.998 |
|  | 3 | >0; ≤ 0.5 | 3.359 | 1.122-10.056 | **0.030** | 8.353 | 0.338-27.96 | 0.319 |
|  | 4 | >0.5 | 1.792 | 0.796-4.061 | 0.158 | 15.212 | 1.405-22.280 | **0.015** |
| Cao et al^41^ | 1 | ≤-2.6 | 1 | (Reference) |  | 1 | (Reference) |  |
|  | 2 | >-2.6; ≤-1.6 | 0.908 | 0.199-4.155 | 0.901 | 2.865 | 0.392-20.972 | 0.299 |
|  | 3 | >-1.6; ≤-0.5 | 1.205 | 0.320-4.536 | 0.782 | 2.592 | 0.356-18.902 | 0.347 |
|  | 4 | >-0.5 | 1.850 | 0.515-6.644 | 0.345 | 5.024 | 0.784-32.194 | 0.089 |
| Chang et al^27^ | 1 | ≤-0.92 | 1 | (Reference) |  | 1 | (Reference) |  |
|  | 2 | -0.91; -0.62 | 0.490 | 0.088-2.729 | 0.420 | 13.768 | 0.297-86.348 | 0.262 |
|  | 3 | -0.61; -0.26 | 0.618 | 0.075-5.120 | 0.660 | 19.356 | 0-Inf. | 0.998 |
|  | 4 | >- 0.26 | 1.153 | 0.665-3.440 | 0.323 | 10.710 | 1.010-15.371 | **0.048** |
| Conci et al^18^ | 1 | ≤ -1.35 | 1 | (Reference) |  | 1 | (Reference) |  |
|  | 2 | >-1.35; ≤ -1 | 2.638 | 0.841-8.270 | 0.096 | 3.392 | 0.532-21-614 | 0.196 |
|  | 3 | >-1; ≤ -0.25 | 0.584 | 0.126-2.702 | 0.491 | 1.284 | 0.106-15.590 | 0.845 |
|  | 4 | >-0.25 | 2.068 | 0.796-5.377 | 0.136 | 6.287 | 1.341-29.478 | **0.020** |
| Fang et al^16^ | 1 | <-0.82 | 1 | (Reference) |  | 1 | (Reference) |  |
|  | 2 | ≥-0.82; < -0.57 | 0.505 | 0.091-2.798 | 0.434 | 4.855 | 0.276-85.500 | 0.280 |
|  | 3 | ≥-0.57 | 1.416 | 0.627-3.200 | 0.403 | 3.299 | 0.817-13.243 | 0.094 |
| Fortea-Sanchis et al^9^ | 1 | <-2 | 1 | (Reference) |  | 1 | (Reference) |  |
|  | 2 | ≥-2; ≤ -1 | 0.888 | 0.271-2.917 | 0.845 | 1.037 | 0.173-6.208 | 0.969 |
|  | 3 | >-1 | 1.188 | 0.356-3.960 | 0.780 | 3.055 | 0.603-15-465 | 0.177 |
| He et al^37^ | 1 | <-3 | 1 | (Reference) |  | 1 | (Reference) |  |
|  | 2 | ≥-3; <-2 | 0.809 | 0.144-4.564 | 0.811 | 2.320 | 0.309-17.403 | 0.413 |
|  | 3 | ≥-2; <-1 | 0.795 | 0.214-2.959 | 0.733 | 1.190 | 0.154-9.186 | 0.867 |
|  | 4 | ≥-1; <0 | 0.348 | 0.059-2.051 | 0.243 | 0.996 | 0.067-14.769 | 0.998 |
|  | 5 | ≥ 0 | 1.337 | 0.351-5.091 | 0.670 | 5.980 | 0.934-38.301 | 0.060 |
| Huang et al^19^ | 1 | <-1 | 1 | (Reference) |  | 1 | (Reference) |  |
|  | 2 | ≥-1; <0 | 0.487 | 0.116-2.052 | 0.327 | 0.896 | 0.085-9.438 | 0.927 |
|  | 3 | ≥0; <1 | 2.187 | 0.813-5.881 | 0.121 | 3.073 | 0.554-17.049 | 0.199 |
|  | 4 | ≥1; <2 | 1.048 | 0.358-3.069 | 0.932 | 6.804 | 0.818-56.585 | 0.076 |
|  | 5 | ≥2 | 4.328 | 1.071-17.479 | **0.040** | 55.145 | 3.762-808.277 | **0.003** |
| Jian-Hui et al^24^ | 1 | ≤-1.5 | 1 | (Reference) |  | 1 | (Reference) |  |
|  | 2 | >-1.5; ≤-1 | 1.714 | 0.549-5.347 | 0.353 | 1.628 | 0.240-11.049 | 0.618 |
|  | 3 | >-1; ≤0 | 0.497 | 0.102-2.248 | 0.364 | 0.907 | 0.073-11.241 | 0.940 |
|  | 4 | >0 | 2.072 | 0.771-5.567 | 0.149 | 6.134 | 1.276-29.489 | **0.024** |
| Lee et al^21^ | 1 | ≤-4 | 1 | (Reference) |  | 1 | (Reference) |  |
|  | 2 | >-4; ≤ -2.5 | 16.343 | 0-Inf | 0.997 | 3.100 | 0-Inf | 0.999 |
|  | 3 | >-2.5; ≤ -2 | 4.243 | 0-Inf | 0.996 | 14.580 | 0-Inf | 0.999 |
|  | 4 | >-2; ≤ -0.5 | 14.516 | 0-Inf | 0.996 | 5.403 | 0-Inf | 0.999 |
|  | 5 | >-0.5 | 26.395 | 0-Inf | 0.997 | 12.908 | 0-Inf | 0.999 |
|  |  |  |  |  |  |  |  |  |
|  |  |  |  |  |  |  |  |  |
|  |  |  |  |  |  |  |  |  |
| Persiani et al^45^ | 1 | ≤-1.36 | 1 | (Reference) |  | 1 | (Reference) |  |
|  | 2 | -1.35; -0.53 | 1.637 | 0.552-4.862 | 0.375 | 3.923 | 0.695-22.138 | 0.122 |
|  | 3 | ≥-0.52 | 2.196 | 0.858-5.616 | 0.101 | 4.560 | 0.994-20.922 | 0.051 |
| Ramacciato et al^38^* | 1 | <-0.005 | 1 | (Reference) |  | 1 | (Reference) |  |
|  | 2 | ≥-0.005; <0.012 | 3.507 | 0-Inf | 0.997 | 4.355 | 0-Inf | 0.998 |
|  | 4 | ≥0.026 | 5.994 | 1.051-4.629 | **0.037** | 14.652 | 1.509-19.241 | **0.009** |
| Riediger et al^15^ | 1 | < -1 | 1 | (Reference) |  | 1 | (Reference) |  |
|  | 2 | -1; -0.500 | 11.858 | 0.075-2.330 | 0.319 | 8.861 | 0.198-53.659 | 0.408 |
|  | 3 | -0.499; -0.001 | 10.198 | 0.053-3.410 | 0.420 | 4.740 | 0-Inf | 0.998 |
|  | 4 | ≥0 | 5.964 | 0.692-3.762 | 0.268 | 14.203 | 1.309-20.842 | **0.019** |
| Song et al^28^ | 1 | ≤-2.51 | 1 | (Reference) |  | 1 | (Reference) |  |
|  | 2 | >-2.51; ≤ -1.68 | 1.106 | 0.233-5.258 | 0.899 | 4.983 | 0.675-36.096 | 0.116 |
|  | 3 | >-1.68; ≤ -0.51 | 1.245 | 0.328-4.731 | 0.748 | 1.983 | 0.286-13.747 | 0.488 |
|  | 4 | >-0.51; ≤ 0.73 | 2.063 | 0.519-8.202 | 0.304 | 2.405 | 0.274-21.118 | 0.429 |
|  | 5 | >0.73 | 1.922 | 0.475-7.786 | 0.360 | 13.695 | 1.642-111.186 | **0.016** |
| Sun et al^10^ | 1 | ≤-1.5 | 1 | (Reference) |  | 1 | (Reference) |  |
|  | 2 | -1.49; -1.000 | 1.713 | 0.549-5.346 | 0.354 | 4.618 | 0.252-11.441 | 0.586 |
|  | 3 | -0.999; -0.500 | 0.517 | 0.086-3.100 | 0.471 | 10.593 | 0.206-73.644 | 0.364 |
|  | 4 | -0.499; 0 | 0.468 | 0.055-3.960 | 0.486 | 3.429 | 0-Inf | 0.999 |
|  | 5 | >0 | 2.079 | 0.770-5.610 | 0.148 | 19.042 | 1.468-33.426 | **0.015** |
| Tang et al^5^ | 1 | ≤-0.9 | 1 | (Reference) |  | 1 | (Reference) |  |
|  | 2 | >-0.9; ≤-0.1 | 0.476 | 0.115-1.970 | 0.305 | 0.986 | 0.093-10.439 | 0.990 |
|  | 3 | >-0.1 | 1.684 | 0.726-3.906 | 0.225 | 4.777 | 1.216-18.767 | **0.025** |
| Toth et al^39^ | 1 | <-1.125 | 1 | (Reference) |  | 1 | (Reference) |  |
|  | 2 | -1.125; -0.251 | 1.656 | 0.549-4.999 | 0.371 | 3.105 | 0.530-18.178 | 0.209 |
|  | 3 | -0.250; 0.749 | 3.183 | 1.053-9.662 | **0.040** | 4.972 | 0.808-30.612 | 0.083 |
|  | 4 | ≥0.750 | 2.194 | 0.800-6.018 | 0.127 | 13.631 | 1.979-93.862 | **0.008** |
|  |  |  |  |  |  |  |  |  |
|  |  |  |  |  |  |  |  |  |
|  |  |  |  |  |  |  |  |  |
|  |  |  |  |  |  |  |  |  |
| Wang et al^33^ | 1 | <-2.2 | 1 | (Reference) |  | 1 | (Reference) |  |
|  | 2 | -2.2; <-1.1 | 0.543 | 0.138-2.132 | 0.382 | 20.824 | 0-Inf | 0.998 |
|  | 3 | -1.1; <0 | 0.842 | 0.213-3.331 | 0.806 | 6.198 | 0.351-14.806 | 0.388 |
|  | 4 | 0; <1.1 | 1.419 | 0.397-5.071 | 0.590 | 7.201 | 0.397-17.700 | 0.315 |
|  | 5 | ≥1.1 | 1.832 | 0.454-7.392 | 0.395 | 6.270 | 3.097-171.267 | **0.002** |
| Wang et al^44^ | 1 | ≤-2.5 | 1 | (Reference) |  | 1 | (Reference) |  |
|  | 2 | >-2.5; ≤ -1 | 1.408 | 0.402-4.925 | 0.593 | 2.586 | 0.446-14.999 | 0.290 |
|  | 3 | >-1; ≤ 0.9 | 1.325 | 0.319-5.496 | 0.698 | 3.459 | 0.443-27.002 | 0.237 |
|  | 4 | >0.9 | 2.184 | 0.536-8.891 | 0.275 | 15.468 | 1.833-130.527 | **0.012** |
| Wu et al^43^ | 1 | ≤-1.46 | 1 | (Reference) |  | 1 | (Reference) |  |
|  | 2 | -1.45; -1.17 | 0.897 | 0.175-4.590 | 0.900 | 4.688 | 0-Inf | 0.999 |
|  | 3 | -1.16; -0.73 | 1.753 | 0.522-5.885 | 0.364 | 8.283 | 0.437-21.248 | 0.260 |
|  | 4 | ≥ -0.72 | 1.849 | 0.705-4.849 | 0.212 | 12.798 | 1.007-21.999 | **0.049** |
| Xu et al^12^ | 1 | ≤-2.8 | 1 | (Reference) |  | 1 | (Reference) |  |
|  | 2 | >-2.8; ≤ -1.60 | 0.671 | 0.146-3.077 | 0.607 | 2.160 | 0.300-15.555 | 0.444 |
|  | 3 | >-1.60; ≤ -0.31 | 1.007 | 0.269-3.768 | 0.992 | 1.981 | 0.266-14.758 | 0.504 |
|  | 4 | >-0.31 | 1.582 | 0.437-5.733 | 0.485 | 5.486 | 0.870-34.607 | 0.070 |
| Xu et al^40^ | 1 | <-1 | 1 | (Reference) |  | 1 | (Reference) |  |
|  | 2 | -1.000; -0.501 | 0.399 | 0.071-2.247 | 0.297 | 8.106 | 0.183-48.438 | 0.442 |
|  | 3 | -0.500; -0.001 | 0.425 | 0.053-3.421 | 0.422 | 3.777 | 0-Inf | 0.998 |
|  | 4 | 0; 0.499 | 2.218 | 0.731-6.725 | 0.159 | 7.505 | 0.287-26.573 | 0.379 |
|  | 5 | ≥0.500 | 1.408 | 0.563-3.524 | 0.465 | 18.354 | 1.563-29.169 | **0.011** |
| Yang et al^36^ | 1 | ≤-1.43 | 1 | (Reference) |  | 1 | (Reference) |  |
|  | 2 | >-1.43; ≤ -1.20 | 0.541 | 0.061-4.790 | 0.581 | 17.763 | 0-Inf | 0.998 |
|  | 3 | >-1.20; ≤ -0.69 | 2.276 | 0.720-7.195 | 0.161 | 8.794 | 0.467-22.387 | 0.234 |
|  | 4 | >-0.69 | 1.990 | 0.762-5.197 | 0.160 | 13.648 | 1.082-23.293 | **0.040** |
| Zhou et al^14^ | 1 | ≤-1 | 1 | (Reference) |  | 1 | (Reference) |  |
|  | 2 | -0.999; -0.500 | 0.418 | 0.076-2.314 | 0.318 | 2.524 | 0.157-40.640 | 0.514 |
|  | 3 | -0.499; 0.500 | 1.317 | 0.465-3.731 | 0.605 | 0.878 | 0.090-8.592 | 0.911 |
|  | 4 | >0.500 | 1.395 | 0.564-3.447 | 0.471 | 6.515 | 1.502-28.263 | **0.012** |

**TABLE S4** C-Statistic of LN Classifications and comparison with the N-Classification with all cases included. None of the investigated cut-off values for novel LN classification systems showed a clear discriminative superiority over the N category in MTC patients with regard to OS.

LN Classification C-Statistic Standard Error C difference P value

N grading 0.8167136 0.0298483 0.0000000 N/A

LODDS Xu 2017^12^ 0.8063083 0.0367554 -0.0104054 0.7561730

LODDS Yang 2017^36^ 0.8189898 0.0313083 0.0022762 0.4238737

LODDS Fortea Sanchis 2018^9^ 0.8001301 0.0376058 -0.0165836 0.8868914

LODDS He 2018^37^ 0.8020811 0.0397740 -0.0146326 0.7624000

LODDS Ramacciato 2017^38^ 0.8110774 0.0371194 -0.0056362 0.6436910

LODDS Toth 2017^39^ 0.8180143 0.0344282 0.00130070 0.4604598

LODDS Zhou 2016^14^ 0.7975287 0.0398650 -0.0191849 0.8340036

LODDS Sun 2010^10^ 0.8117277 0.0379057 -0.0049859 0.6014954

LODDS Xu 2013^40^ 0.8057663 0.0392651 -0.0109473 0.7098075

LODDS Riediger 2016^15^ 0.8046824 0.0395033 -0.0120312 0.7269494

LODDS Fang 2017^16^ 0.7931931 0.0399497 -0.0235205 0.8896789

LODDS Conci 2017^18^ 0.8157381 0.0345705 -0.0009755 0.5232119

LODDS Huang 2016^19^ 0.8108606 0.0383630 -0.0058530 0.6130124

LODDS Calero 2015^20^ 0.8021895 0.0356976 -0.0145242 0.8757589

LODDS Amini 2015^46^ 0.8103187 0.0388708 -0.0063950 0.6449022

LODDS Lee 2016^21^ 0.8095599 0.0370178 -0.0071537 0.6874621

LODDS Cao 2016^41^ 0.8055495 0.0359004 -0.0111641 0.7984238

LODDS Amini 2016^42^ 0.8075005 0.0381346 -0.0092131 0.7034777

LODDS Bagante 2016^23^ 0.8154130 0.0341760 -0.0013007 0.5427228

LODDS Jian-Hui 2016^24^ 0.8118361 0.0380007 -0.0048775 0.5988692

LODDS Wu 2015^43^ 0.8063083 0.0354208 -0.0104054 0.7928223

LODDS Wang 2013^44^ 0.8093432 0.0354755 -0.0073705 0.7335314

LODDS Chang 2012^27^ 0.8003468 0.0397578 -0.0163668 0.7974997

LODDS Song 2011^28^ 0.8065250 0.0357094 -0.0101886 0.7963047

LODDS Persiani 2012^45^ 0.8122697 0.0345042 -0.0044440 0.6438571

LODDS Wang 2008^33^ 0.8135703 0.0377429 -0.0031433 0.5763051

LODDS Cao 2019^32^ 0.8148710 0.0366486 -0.0018426 0.5482138

LODDS Tang 2021^5^ 0.8033817 0.0399646 -0.0133319 0.7453799

LNR Xu 2017^12^ 0.8129200 0.0330711 -0.0037936 0.6631471

LNR Arslan 2014^11^ 0.8168220 0.0328552 0.0001084 0.4960952

LNR Malleo 2015^13^ 0.8180143 0.0314483 0.0013007 0.4324708

LNR Sun 2010^10^ 0.8186646 0.0332824 0.0019510 0.4237854

LNR Zhou 2016^14^ 0.8048992 0.0368797 -0.0118144 0.8106561

LNR Riediger 2016^15^ 0.8243009 0.0313618 0.0075873 0.2578422

LNR Fang 2017^16^ 0.8057663 0.0348852 -0.0109473 0.8308237

LNR Wang 2011^33^ 0.8158465 0.0338791 -0.0008671 0.5332124

LNR Conci 2017^18^ 0.8117277 0.0327535 -0.0049859 0.7791325

LNR Huang 2016^19^ 0.8037069 0.0395701 -0.0130067 0.7437213

LNR Calero 2015^20^ 0.8136787 0.0325273 -0.0030349 0.6682613

LNR Lee 2016^21^ 0.8215912 0.0306051 0.0048775 0.3555201

LNR Kim 2015^22^ 0.8180143 0.0338207 0.0013007 0.4515093

LNR Bagante 2016^23^ 0.8188814 0.0339893 0.0021678 0.4369827

LNR Jian-Hui 2016^24^ 0.8119445 0.0327228 -0.0047691 0.7695028

LNR Smith 2014^25^ 0.8198569 0.0318371 0.0031433 0.3496378

LNR Liu 2013^26^ 0.8118361 0.0333331 -0.0048775 0.7184296

LNR Chang 2012^27^ 0.8140039 0.0332275 -0.0027097 0.5940211

LNR Song 2011^28^ 0.8101019 0.0329761 -0.0066117 0.7862600

LNR La Torre 2014^29^ 0.8221331 0.0295821 0.0054195 0.2590595

LNR Agnes 2019^30^ 0.8075005 0.0343394 -0.0092131 0.7701778

LNR Rosenberg 2008^31^ 0.8159549 0.0327412 -0.0007587 0.5369884

LNR Cao 2019^32^ 0.8121613 0.0327302 -0.0045524 0.7588714

LNR Fortea-Sanchis 2018^9^ 0.8065250 0.0376145 -0.0101886 0.7403542

LNR Wang 2008^17^ 0.8043573 0.0397684 -0.0123564 0.7299586

LNR Chen 2020^34^ 0.8189898 0.0340808 0.0022762 0.4276804

LNR Jiang 2017^35^ 0.8071754 0.0361087 -0.0095383 0.7722079

LNR Rozenblat 2020^4^ 0.8048992 0.0346978 -0.0118144 0.8456483

**TABLE S5** C-statistic of LN classifications and comparison with the N category in patients without metastatic disease.

LN Classification C-Statistic Standard Error C difference P value

N grading 0.8225675 0.0472312 0.0000000 N/A

LODDS Xu 2017^12^ 0.7710548 0.0755182 -0.0515127 0.8497607

LODDS Yang 2017^36^ 0.8160262 0.0571322 -0.0065413 0.5621575

LODDS Fortea-S 2018^9^ 0.7714636 0.0719793 -0.0511038 0.8412838

LODDS He 2018^37^ 0.7551104 0.0842631 -0.0674571 0.8693049

LODDS Ramac. 2017^38^ 0.7624693 0.0726457 -0.0600981 0.8809524

LODDS Toth 2017^39^ 0.7976288 0.0663156 -0.0249387 0.6971634

LODDS Zhou 2016^14^ 0.7804579 0.0742260 -0.0421096 0.7706195

LODDS Sun 2010^10^ 0.8037612 0.0684131 -0.0188062 0.6435958

LODDS Xu 2013^40^ 0.7910875 0.0745034 -0.0314800 0.7108228

LODDS Riediger 2016^15^ 0.7886345 0.0719926 -0.0339330 0.7381622

LODDS Fang 2017^16^ 0.7788226 0.0706034 -0.0437449 0.8064550

LODDS Conci 2017^18^ 0.7681930 0.0714563 -0.0543745 0.8590452

LODDS Huang 2016^19^ 0.7624693 0.0740322 -0.0600981 0.8679763

LODDS Calero 2015^20^ 0.7735078 0.0759834 -0.0490597 0.8296736

LODDS Amini 2015^46^ 0.7514309 0.0770892 -0.0711365 0.9020595

LODDS Lee 2016^21^ 0.7825020 0.0700926 -0.0400654 0.8158679

LODDS Cao 2016^41^ 0.7739166 0.0760803 -0.0486509 0.8357432

LODDS Amini 2016^42^ 0.7677841 0.0744992 -0.0547833 0.8574693

LODDS Bagante 2016^23^ 0.7951758 0.0643109 -0.0273917 0.7215464

LODDS Jian-Hui 2016^24^ 0.7600164 0.0731733 -0.0625511 0.8838899

LODDS Wu 2015^43^ 0.8180703 0.0560451 -0.0044971 0.5437594

LODDS Wang 2013^44^ 0.7878168 0.0720551 -0.0347506 0.7589787

LODDS Chang 2012^27^ 0.7959935 0.0687146 -0.0265740 0.6999573

LODDS Song 2011^28^ 0.7800491 0.0772996 -0.0425184 0.7797437

LODDS Persiani 2012^45^ 0.8041701 0.0623766 -0.0183974 0.6594232

LODDS Wang 2008^33^ 0.8405560 0.0507879 0.0179886 0.3295521

LODDS Cao 2019^32^ 0.7739166 0.0709511 -0.0486509 0.8294853

LODDS Tang 2021^5^ 0.7542927 0.0750104 -0.0682747 0.9014500

LNR Xu 2017^12^ 0.8262469 0.0540502 0.0036795 0.4336891

LNR Arslan 2014^11^ 0.8086672 0.0652372 -0.0139002 0.6233515

LNR Malleo 2015^13^ 0.8286999 0.0469721 0.0061325 0.2358908

LNR Sun 2010^10^  0.8233851 0.0563661 0.0008177 0.4871229

LNR Zhou 2016^14^ 0.7792314 0.0708900 -0.0433361 0.7980326

LNR Riediger 2016^15^ 0.8401472 0.0527728 0.0175797 0.3336173

LNR Fang 2017^16^ 0.7649223 0.0667602 -0.0576451 0.9038285

LNR Wang 2011^33^ 0.8282911 0.0543166 0.0057236 0.4100669

LNR Conci 2017 ^18^ 0.8242028 0.0493648 0.0016353 0.4315920

LNR Huang 2016^19^ 0.7681930 0.0713827 -0.0543745 0.8528188

LNR Calero 2015^20^ 0.8323794 0.0534174 0.0098119 0.3492925

LNR Lee 2016^21^ 0.8004906 0.0611369 -0.0220769 0.6901386

LNR Kim 2015^22^ 0.8237939 0.0560279 0.0012265 0.4805109

LNR Bagante 2016^23^ 0.8266558 0.0572614 0.0040883 0.4432268

LNR Jian-Hui 2016^24^ 0.8299264 0.0512371 0.0073590 0.3377996

LNR Smith 2014^25^ 0.8315617 0.0622631 0.0089943 0.4095860

LNR Liu 2013^26^ 0.8237939 0.0513176 0.0012265 0.4685219

LNR Chang 2012^27^ 0.8107114 0.0535297 -0.0118561 0.6190662

LNR Song 2011^28^ 0.8286999 0.0552749 0.0061325 0.4173702

LNR La Torre 2014^29^ 0.8532298 0.0417541 0.0306623 0.0631408

LNR Agnes 2019^30^ 0.8262469 0.0513939 0.0036795 0.4099369

LNR Rosenberg 2008^31^ 0.8299264 0.0525142 0.0073590 0.3775875

LNR Cao 2019^32^ 0.8233851 0.0496329 0.0008177 0.4689238

LNR Fortea-S. 2018^9^ 0.7579722 0.0747675 -0.0645953 0.8869034

LNR Wang 2008^17^ 0.7514309 0.0760152 -0.0711365 0.9055634

LNR Chen 2020^34^ 0.7865904 0.0656767 -0.0359771 0.7828458

LNR Jiang 2017^35^ 0.7702371 0.0651667 -0.0523303 0.8637228

LNR Rozenblat 2020^4^ 0.7665576 0.0631140 -0.0560098 0.9160844
